# Concordance Between Resident and Attending Radiologist in Reporting Pneumothorax on Intensive Care Unit and Emergency Room Chest Radiographs

**DOI:** 10.7759/cureus.29672

**Published:** 2022-09-27

**Authors:** Anam Hafeez, Naila Nadeem, Junaid Iqbal, Aneeqa Qureshi, Asad Shakeel, Uffan Zafar

**Affiliations:** 1 Radiology, Civil Hospital Sanghar, Sanghar, PAK; 2 Radiology, Aga Khan University Hospital, Karachi, PAK; 3 Radiology, Dr. Ziauddin Hospital, Karachi, PAK; 4 Diagnostic Radiology, Patel Hospital, Karachi, PAK; 5 Radiology, Bahawal Victoria Hospital, Quaid-e-Azam Medical College, Bahawalpur, PAK

**Keywords:** emergency room (er), intensive care unit (icu), radiograph, radiologist, resident, pneumothorax

## Abstract

Introduction

Pneumothorax is a common medical emergency and has potentially life-threatening consequences, so it is important for radiology residents and consultants to know its radiographic appearance so that timely diagnosis and appropriate management can be done. Patients with pneumothorax have nonspecific complaints, and clinical examinations are not confirmatory. The chest X-ray is easily available and has high accuracy in the detection of pneumothorax. The aim of this study is to determine the agreement between the on-call radiology resident and the attending radiologist in the diagnosis of pneumothorax on chest radiographs.

Materials and methods

This cross-sectional study was performed in the Department of Radiology at Aga Khan University Hospital, Karachi. After approval from the ethical review committee (ERC), the study was carried out. A total of 174 patients were included in the study. The resident interpreting the radiograph commented on the pneumothorax and recorded it on the “Comments” section of the picture archiving and communication system (PACS). Further entries were made in the department’s “Panic Logbook.” Subsequently, the final report by the attending radiologist was tallied, and the decision of both the resident and the attending radiologist regarding the presence or absence of pneumothorax was compared for interobserver agreement.

Results

Of the 174 patients, 139 (79.9%) were male and 35 (20.1%) were female. The mean age of the patients was 45.6 ± 12.4 years. Pneumothorax was reported by the resident in 164 (94.25%) cases, while the attending radiologist reported it in 167 (96%) cases. The remaining 4% of cases were ultimately diagnosed on a CT scan of the chest performed at the request of the primary team; they were too small to be detected on a chest radiograph.

The most common side involved was the right side, with 112 (64.4%) cases, followed by the left side with 55 (31.6%) and both sides with five (2.9%), while in two cases, pneumothorax was not reported by the resident and the attending radiologist. The position of the pneumothorax was as follows: apex in 80 (46%), base in 56 (32.2%), and along the lateral border of the lung in 93 (53.4%). Concordance between the resident and the radiologist was found to be 92.5% (kappa = 0.20; p = 0.008). Stratification for age, gender, the position of pneumothorax, and the level of residency was also carried out.

Conclusion

In our setting, there was a high level of agreement (92.5%) between the resident and the attending radiologist in reporting pneumothorax on chest radiographs (kappa = 0.20; p = 0.008).

## Introduction

Pneumothorax is a medical emergency in which there is a collapse of the lung and the accumulation of air in the pleural cavity. Pneumothorax can be spontaneous or traumatic [1]. Spontaneous pneumothorax is further classified into primary and secondary pneumothoraces [2]. In primary spontaneous pneumothorax, patients develop pneumothorax without apparent lung disease [3]. It usually affects young, healthy men. Although there is an absence of underlying lung disease, rupture of subpleural blebs, cysts, or bullae is likely to play a role in the development of spontaneous pneumothorax [4]. Women are 4-10 times less likely to suffer from it [5]. Smoking can play an important role in the development of primary spontaneous pneumothorax [6]. There is a 12% risk of developing a pneumothorax in smokers versus 0.1% in nonsmoker men. The same trend is present in women but to a lesser extent [7]. Primary spontaneous pneumothorax is initially managed by chest tube placement or by simple aspiration of air from the pleural cavity [8]. In secondary spontaneous pneumothorax, there is an underlying lung disease, and it often presents as a potentially life-threatening condition, requiring immediate intervention [9]. The majority of the patients with secondary pneumothorax are elderly and have underlying chronic obstructive pulmonary disease (COPD) or interstitial pneumonia [1].

Pneumothorax can also be divided into tension pneumothorax and non-tension pneumothorax. Tension pneumothorax is a medical emergency because of the progressive accumulation of air in the pleural space, leading to circulatory and respiratory failure by the compression of mediastinal structures if not managed immediately, whereas non-tension pneumothorax is not as critical as tension pneumothorax because there is no compression of intrathoracic organs [2]. Iatrogenic pneumothorax is a common complication of many procedures, such as thoracentesis, percutaneous lung biopsy [10], feeding tube placement [11], and mechanical ventilation [12]. Such procedures routinely require additional observation time after the procedure, follow-up radiographic examinations, and occasional hospital admissions.

On radiographs, pneumothorax is seen as a visceral pleural line without distal lung markings. The underlying lung may or may not be completely collapsed. Tension pneumothorax is characterized by a mediastinal shift to the opposite side, diaphragmatic depression, and ipsilateral hyper-expansion [13,14]. There is a 28%-75% sensitivity of supine chest X-ray for the diagnosis of pneumothorax in patients with blunt trauma and has a specificity of 100% [15]. Pneumothorax can often be overlooked, especially by junior radiology residents during on-call hours. It is known that the interpretation skills of chest radiography increase with experience in terms of diagnostic accuracy. To the best of our knowledge, no study on concordance rates between trainees and radiologists for pneumothorax has been conducted in Pakistan. Practical recommendations for improvement in training programs can be made if there is a poor interobserver agreement for pneumothorax. This could result in improved patient care and better clinical outcomes.

## Materials and methods

This was a cross-sectional study performed in the Department of Radiology at Aga Khan University Hospital, Karachi, from 01-07-2020 to 31-12-2020, after approval from the ethical review committee (ERC). No additional imaging or procedures needed to be performed for the purpose of this study. Therefore, no additional cost or radiation hazards were expected from this study. Finally, strict confidentiality and anonymity of all subjects were maintained. After taking informed consent, a total of 174 radiographs of patients from the emergency room (ER) and intensive care units (ICUs) of Aga Khan University Hospital who were labeled positive for pneumothorax by the radiology resident or attending radiologist on chest radiography were included in this study. The images were reviewed by a resident on the picture archiving and communication system (PACS) immediately after the radiographs were carried out. The resident interpreting the radiograph commented on the pneumothorax and recorded it in the “Comments” section of PACS. Further entries were made in the department’s “Panic Logbook.” (Under the supervision of the radiology manager and section head of general radiology, our general radiology section keeps a logbook of the panic findings. The logbook is maintained on a monthly basis with identification details of patients and panic findings and is checked regularly through audits.) After the entry of the panic finding, the final report by the attending radiologist was tallied, and the decision of both the resident and the attending radiologist on the presence or absence of pneumothorax was compared for interobserver agreement. A proforma was used to record the data. The Statistical Package for the Social Sciences (SPSS) version 20.0 software (IBM Corp., Armonk, NY, USA) was used to enter the data. Age and other quantitative data were expressed as mean ± standard deviation (SD). The resident and radiologist calculated frequencies and percentages for qualitative data such as gender, side, and pneumothorax detection. Kappa statistics were applied to assess the concordance between both readers. A p value of less than or equal to 0.05 was considered significant. Effect modifiers such as age, gender, the position of pneumothorax, and the level of residency were controlled using stratification. Post-stratification data analysis was performed. A Kappa test was applied to measure the extent of interobserver variation between the resident and the attending radiologist.

## Results

**Figure 1:**
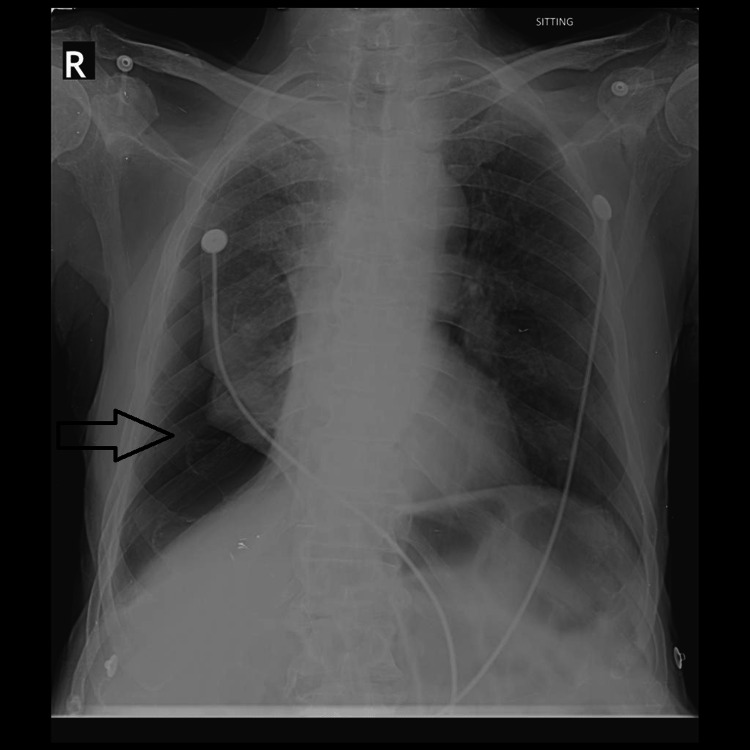
Right-sided pneumothorax (black arrow) reported by both the resident and the attending radiologist

**Figure 2:**
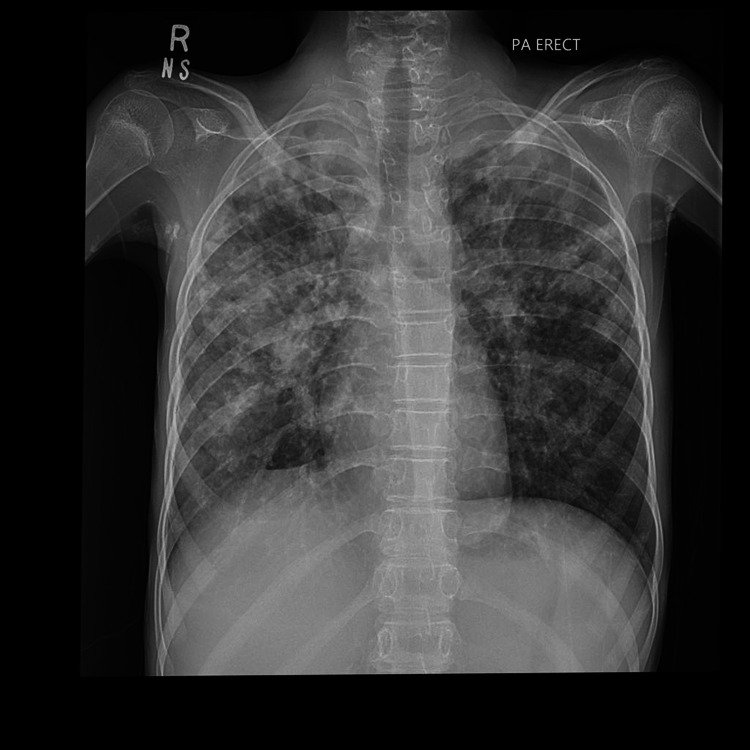
No pneumothorax reported by both the resident and the attending radiologist

**Figure 3:**
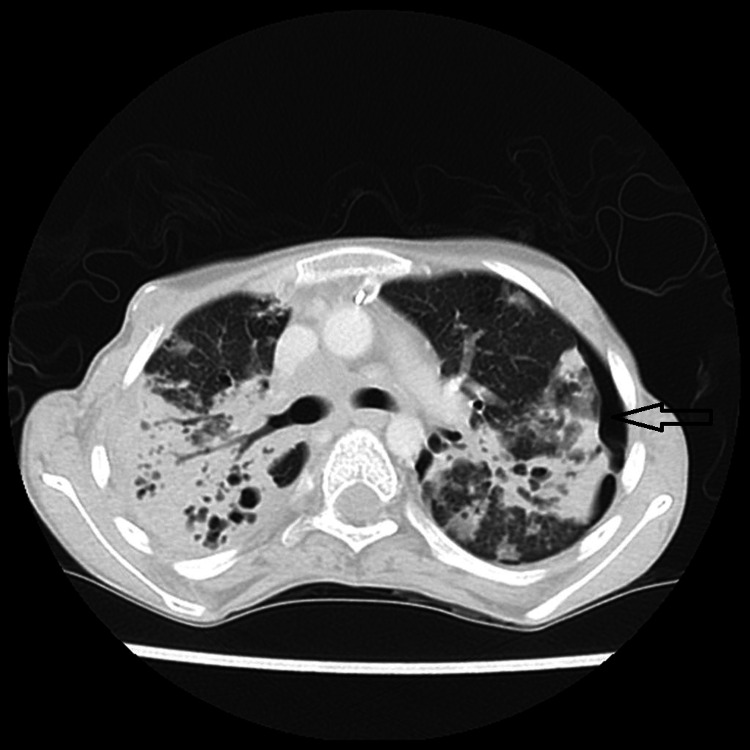
Subtle left-sided pneumothorax (black arrow) not detected on the previous radiograph by both the resident and the attending radiologist

Of the 174 patients, 139 (79.9%) were male and 35 (20.1%) were female. The mean age of the patients was 45.6 ± 12.4 years. The resident reported pneumothorax in 164 (94.25%) cases, while the attending radiologist reported it in 167 (96%) cases (Figure 1, Table 1). The most common side involved was the right side (Figure 1), with 112 (64.4%) cases, followed by the left side with 55 (31.6%) and both sides with five (2.9%), while in two cases, pneumothorax was not reported by the resident and the attending radiologist; both were ultimately diagnosed on a CT scan of the chest performed at the request of the primary team as they were too small to be detected on chest radiograph (Figures 2, 3). The position of the pneumothorax was as follows: apex in 80 (46%), base in 56 (32.2%), and along the lateral border of the lung in 93 (53.4%). Concordance between the resident and the radiologist was found to be 92.5% (kappa = 0.197; p = 0.008) (Table 1). Stratification for age, gender, the position of pneumothorax, and the level of residency was also carried out (Tables 2-5).

**Table 1:** Concordance between the resident and the radiologist kappa = 0.197 p = 0.008 Concordance = 92.5% (a + d / a + d + b + c × 100)

Pneumothorax reported by the resident	Pneumothorax reported by the radiologist		Total
	Yes	No	
Yes	159 (a)	5 (b)	164
No	8 (c)	2 (d)	10
Total	167	7	174

**Table 2:** Stratification for age

Age (year)	Pneumothorax reported by the resident	Pneumothorax reported by the radiologist		Total	Kappa/p value
		Yes	No		
25-40	Yes	58	2	60	-0.040/0.748
	No	3	0	3	
Total		61	2	63	
41-60	Yes	101	3	104	0.296/0.002
	No	5	2	7	
Total		106	5	111	

**Table 3:** Stratification for gender

Gender	Pneumothorax reported by the resident	Pneumothorax reported by the radiologist		Total	Kappa/p value
		Yes	No		
Male	Yes	127	2	129	0.255/0.001
	No	8	2	10	
Total		135	4	139	
Female	Yes	32	3	35	-
	No	-	-		
Total		32	3	35	

**Table 4:** Stratification for the position of pneumothorax (apex, base, and along the lateral border of the lung)

Position of pneumothorax	Pneumothorax reported by the resident	Pneumothorax reported by the radiologist		Total	Kappa/p value
		Yes	No		
Apex	Yes	72	4	76	-0.053/0.638
	No	4	-	4	
Total		76	4	80	
Base	Yes	87	1	88	-0.028/0.810
	No	4	-	4	
Total		91	1	92	
Along the lateral border of the lung	Yes	72	4	76	-0.026/0.794
	No	4	-	4	
Total		76	4	80	

**Table 5:** Stratification for the level of radiology residents

Level of residents	Pneumothorax reported by the resident	Pneumothorax reported by the radiologist		Total	Kappa/p value
		Yes	No		
First year (PGY-1)	Yes	81	5		0.203/0.048
	No	6	2		
Total		87	7	94	
Second year (PGY-2)	Yes	6	0	6	-
	No	1	0	1	
Total		7	0	7	
Third year (PGY-3)	Yes	26	0	26	-
	No	-	0	-	
Total		26	0	26	
Fourth year (PGY-4)	Yes	46	0	46	-
	No	1	0	1	
Total		47	0	47	

## Discussion

Portable chest radiography is a commonly used imaging modality in patients admitted to intensive care units (ICUs) [16]. However, there remain several technical challenges for the detection and diagnosis of pneumothorax due to limitations such as improper patient positioning, inadequate inspiration, patient non-cooperation, and obscuration of fine anatomical details due to superimposition of anatomical structures, overlying chest tubes, cardiac monitoring devices, and vascular lines. Because transporting ICU patients to the radiology department for radiography is difficult, it is critical to evaluate and formulate strategies for improving sensitivity in the diagnosis of a pneumothorax on portable chest radiography. The initial evaluation of a portable chest radiograph is usually performed by an inexperienced reader, and this may cause a delay in the timely diagnosis of pneumothorax [17].

In teaching and university hospitals, the initial evaluation of diagnostic imaging is usually performed by an on-call radiology resident. A consultant radiologist then reviews those cases the following morning. The experience of working during on-call hours and making independent decisions in challenging cases is essential to completing residents’ training and education. The goal of any residency program is to strike a balance between patient care and maintaining a good educational experience during an overnight call. To ensure the high quality of patient care in the radiology department, the performance of residents must be monitored, analyzed, supervised, and improved [18]. On-call radiology residents are responsible for the preliminary diagnosis of imaging that may impact patient management before the final report by the consultant [19]. Although resident education is important, patient care cannot be compromised. Therefore, analysis of the effect of misinterpretations by the residents on patient care is mandatory [18,19].

Despite all the new developments in radiology, chest radiographs are still the first imaging modality for various diseases because of their rapid results [20]. Chest radiographs are often initially evaluated by clinicians, where the clinicians’ own radiological skills play a vital role. Clinicians decide the management based on their own interpretation without having the opportunity to interact with the radiologists in life-threatening and emergency cases. Several studies have been carried out to compare the diagnostic misinterpretation of radiographs by clinicians as compared to radiologists [21,22]. These studies conclude that clinicians are not interpreting radiographs correctly as radiologists. In the current study, a strong agreement was found between the two observers (kappa = 0.197; p = 0.008). The concordance rate between the resident and the attending radiologist was found to be 92.5%. Our findings are comparable with a study carried out by Cooper et al. [23].

In our study, the overall discrepancy rate between residents and attending radiologists in diagnosing pneumothorax is 7.5%, which is comparable with other published studies that have compared resident and attending radiologist interpretations. Carney et al. [19] found a discrepancy rate of 3.8% between residents and attending radiologists in the interpretation of CT scans and sonography at a trauma center, Wechsler et al. [24] found a discrepancy rate of 1.2% in the interpretation of emergency body CT scans, Wysoki et al. [25] found a discrepancy rate of 1.7% in the head trauma CT scan, and Velmahos et al. [26] found a discrepancy rate of 5% in the interpretation of CT scans in trauma patients. Our discrepancy rate is higher as compared to the abovementioned studies due to different imaging modalities (radiograph versus CT scan). Azapoglu Kaymak et al. [27] concluded that the correct diagnosis of pneumothorax on the radiograph was 83.3%, 75.5%, and 62.5% among senior, middle, and junior residents, respectively. Similar findings were reported in our study, where it was discovered that the postgraduate residents’ residency year had a substantial impact on the accurate diagnosis of pneumothorax on the chest radiograph. As the experience of the residents increased (from PGY-1 to PGY-4), the rate of correct diagnosis of pneumothorax also increased, and the discrepancy rate decreased.

## Conclusions

Pneumothorax is a medical emergency, so its timely and accurate diagnosis is essential for the management of this life-threatening condition. In our setting, there was a high level of agreement (92.5%) between residents and attending radiologists in reporting pneumothorax on emergency room (ER) and intensive care unit (ICU) radiographs (kappa = 0.197; p = 0.008). On the basis of our results, we concluded that during on-call hours, radiology residents can safely identify and diagnose pneumothorax, so its management can be started without delay.
